# ^99^Tc^m^-MDP Imaging of Osteopetrosis: Case Report

**DOI:** 10.1097/MD.0000000000000929

**Published:** 2015-06-05

**Authors:** Li-Chun Zheng, Xiang-Liu OuYang, Gui-Chao Liu, Wen-Jun Zhang, Xiao-Ming Zhang

**Affiliations:** From the Department of Nuclear Medicine, Department of ultrasound, Tangshan Gongren Hospital, Tangshan, Hebei Province, China.

## Abstract

Osteopetrosis, also known as marble bone disease, is a clinically rare genetic disease, which represents a heterogeneous group of rare, inherited bone dysplasias that share the hallmark of abnormally increased bone density caused by osteoclast dysfunction. Hereby, the authors describe a case of osteopetrosis that showed increased diffuse radioactive uptake on whole body bone ^99^Tc^m^-methylene diphosphonate imaging in a 56-year-old man, which increased universal radioactive uptake on craniofacial bone imaging, and enlargement of the limb long bone near the joints with evenly symmetrical enriched distribution of radioactivity. Osteopetrosis was made which based on these features and characteristics shown on 99Tcm-MDP imaging.

Skeletal scintigraphy with ^99^Tc^m^-methylene diphosphonate imaging is helpful to the diagnosis of osteopetrosis. There is a characteristic of osteopetrosis different from other bone metabolic diseases.

## INTRODUTION

Osteopetrosis, also known as marble bone disease, was first reported in 1904 by Albers-Schonberg as a bone developmental disorder with an incidence rate of approximately 1/50 million, which represents a heterogeneous group of rare, inherited bone dysplasias that share the hallmark of abnormally increased bone density caused by osteoclast dysfunction.^1^ We report a case of osteopetrosis imaging of whole body bone ^99^Tc^m^-methylene diphosphonate (MDP) and x-ray in a 56-year-old man.

## PATIENT INFORMATIONG

A 56-year-old male patient has been prone to fracture since his childhood and has experienced poor healing with a history of multiple fractures of the bilateral lower limbs. He underwent surgery for a left mandibular fracture 24 years ago, which exhibited poor healing with morphological abnormalities. He has no family history of related bone disorders.

## CLINICAL FINDINGS AND DIAGNOSTIC ASSESSMENT

The results of a bone density examination were normal. And the blood tests revealed creatine kinase level of 360 U/L (normal range, 25–200 U/L), creatine kinase-MB level of 304 U/L ((normal range, 0–26 U/L), alkaline phosphatase level of 122 U/L (normal range, 30–114 U/L), lipoprotein(a) level of 1323 mg/L (normal range, 0–300 mg/L), and neuron-specific enolization enzymes level of 25.30 ng/mL (normal range, 0–16.3 ng/mL). All other indicators were normal.

**FIGURE 1:**
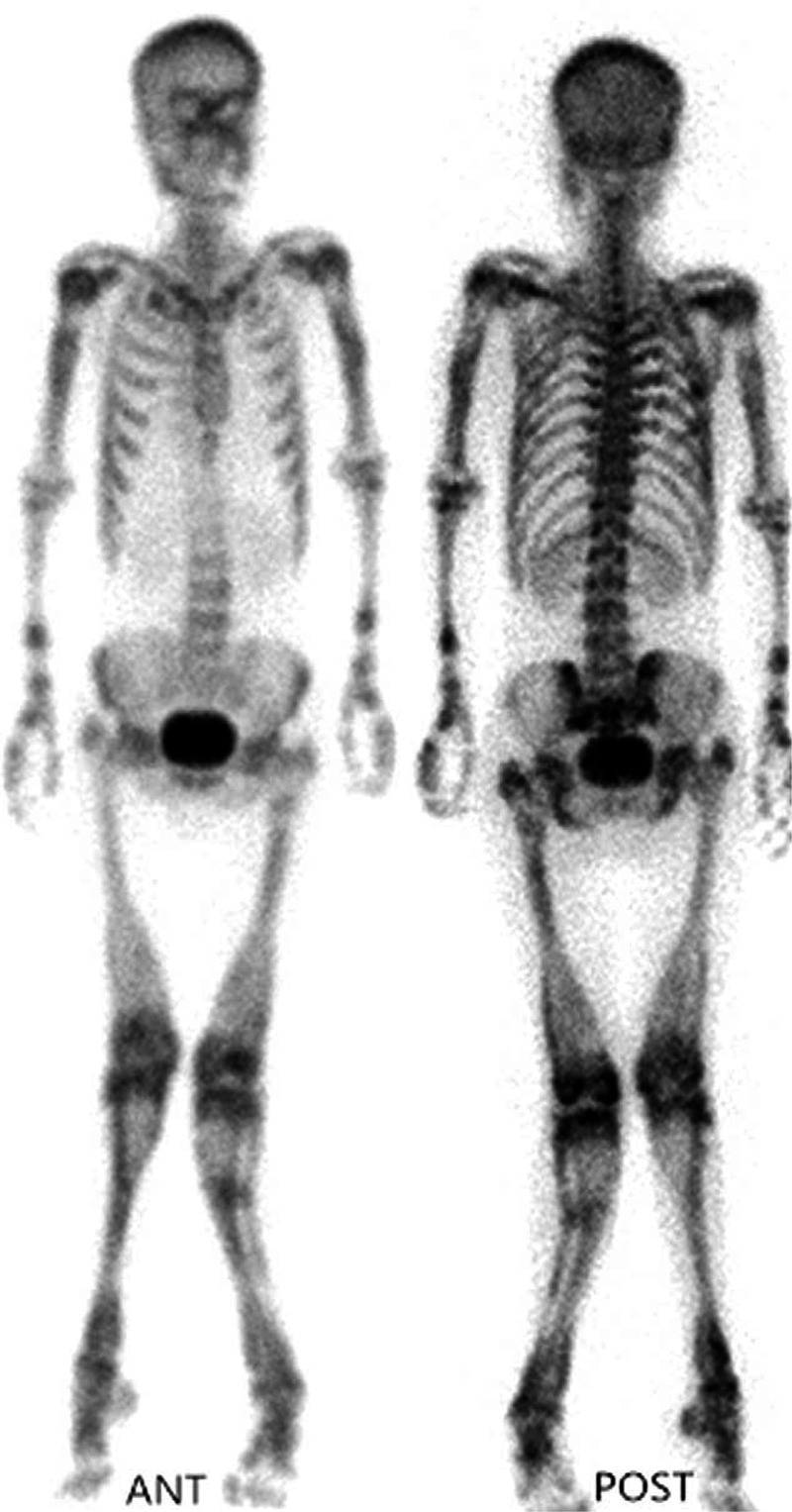
Whole body bone 99Tcm-MDP imaging of patient.

Skeletal scintigraphy was performed approximately 3 hours after intravenous injection of 925 MBq (25 mCi) dose of 99Tcm-MDP. Anterior and posterior views of the entire body were obtained using a dual-head single photon emission computed tomography equipped with low-energy general purpose collimators, which was planar mode of acquisition. ^99^Tc^m^-MDP scanning demonstrated increased diffuse radioactive uptake on whole body bone imaging, increased symmetrical radioactive distribution in the bilateral ilia, and varus of both knees (history of multiple fractures). Intense uptake was noted in the spine, at the epiphyses and metaphyses of the proximal and distal femora, and proximal and distal metaphyses of both tibiae. Another interesting finding on bone scintigraphy was nonvisualization of both kidneys (Figure 1). Osteopetrosis was considered.

**FIGURE 2:**
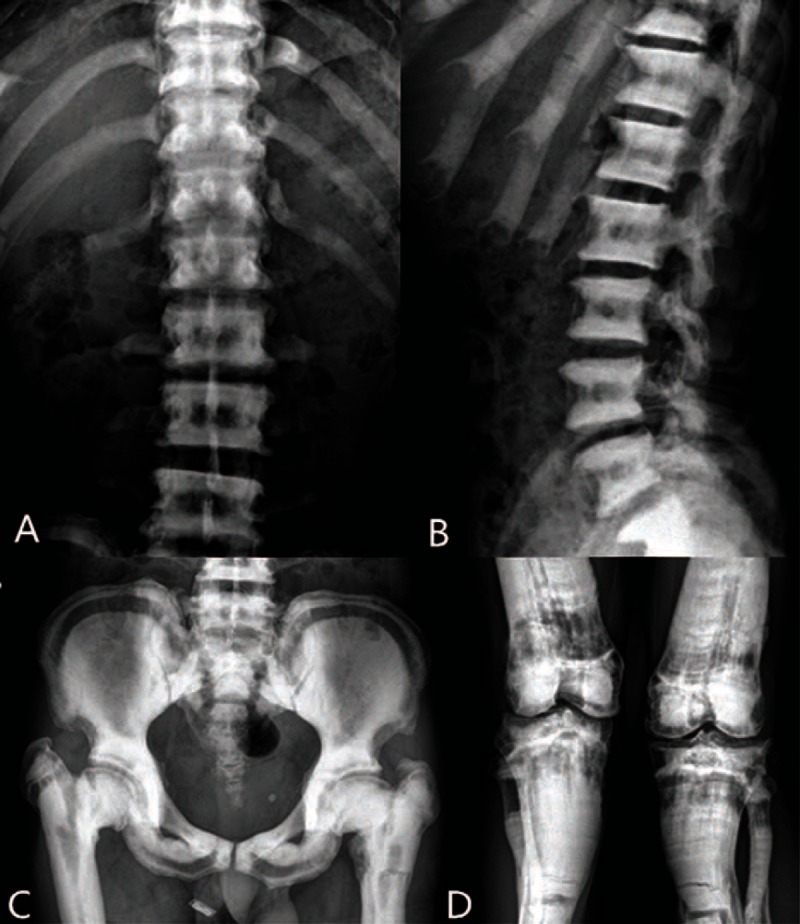
X-ray images of patient's spine, pelvic and both knees.

X-ray images showed increased bone density at the spine and ribs and bone sclerosis, which is a “layer cake” like change in the vertebral body, and a shadow of transverse fracture lines in some ribs (Figure 2A and B). The images showed increased pelvic bone density, which curved strip of a low-density zone at the bilateral ilia, pubis, and ischium. Bilateral femoral neck was shorter, upshift of the femoral shaft, and fracture line shadow on both sides of the proximal femur (Figure 2C). The images showed increased bone mineral density in both knees with bone sclerosis, and the shadow of multiple transverse fracture lines on the upper segment of the bilateral tibia and fibula (Figure 2D). These observations were in line with the characteristic features of osteopetrosis.

## FOLLOW-UP AND OUTCOMES

The patient was discharged after 2 weeks with a symptomatic therapy with corticosteroids and vitamin D.

## DISCUSSION

Osteopetrosis is a heterogeneous group of hereditary diseases of bone resorption, manifested by increased bone mass and bone density.^2–4^ Osteopetrosis may be caused by poor osteoclast function, which being unable to release enough lysosomal enzymes, or the disruption of the physiological balance between osteoblasts and osteoclasts, thereby leading to a bone resorption barrier.^5–7^ Osteopetrosis is divided into two types. First is an infantile type (malignant type), also known as autosomal recessive osteopetrosis. Symptoms often appear in infancy and include progressive anemia and hepatosplenomegaly, skull bone sclerosis causing neurovascular compression showing corresponding symptoms, bone sclerosis prone to fracture, and acute illness, often resulting in death due to severe anemia and recurrent infections. Second is an adult type (benign type), also known as autosomal dominant osteopetrosis. The lesions are mainly located in the skeletal system with minor bone marrow changes and anemia, it is often incidentally revealed by an x-ray examination for other reasons, and the prognosis is good.^8–11^ The patient in this study was adult-type osteopetrosis with a good prognosis who is currently alive.

X-ray imaging plays a role in confirming the diagnosis by showing several characteristic features:  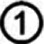 the marble-like bone is generally hyperdense and sclerotic with thickening of the cortical bones, narrowing of the canal, and thickening bone density at the upper and lower edges with reduced density of the middle of the vertebral body, which shows a “layer cake” change;  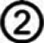 widened bone ends and transverse density shadows in alternating dark and light shades that show a zebra-like pattern;  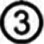 bone within bone; and  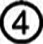 patients are prone to fracture.

Skeletal scintigraphy with ^99^Tc^m^-MDP imaging is helpful to the diagnosis of osteopetrosis. It can show the imaging of whole body bone. Osteopetrosis may show “superscan”. This “superscan” can be related to the rapid and enhanced uptake of tracer by the abnormally thickened cortical bone, resulting in reduced radionuclide excretion by the kidneys. This is characteristic of metabolic bone disease or widespread metastatic bone involvement. But osteopetrosis is different from other bone metabolic diseases, such as intense uptake at the metaphyseal regions of the long bones, and intense uptake was noted in the spine, at the epiphyses and metaphyses of the proximal humeri and distal femora, and proximal and distal metaphyses of both tibiae.^12–16^ The Table 1 is the literature of “osteopetrosis and bone scan”, there are some patterns of presentations. Bone scan shows a diffuse increase in skeletal uptake and symmetrically increased focal uptake in the metaphysic and adjacent diaphysis of multiple long bones, which are typical findings in osteopetrosis. In addition, tomography imaging (single photon emission computed tomography imaging) was no obvious advantage than planar mode acquisition for the diagnosis of osteopetrosis with ^99^Tc^m^-MDP imaging.

**TABLE 1:**
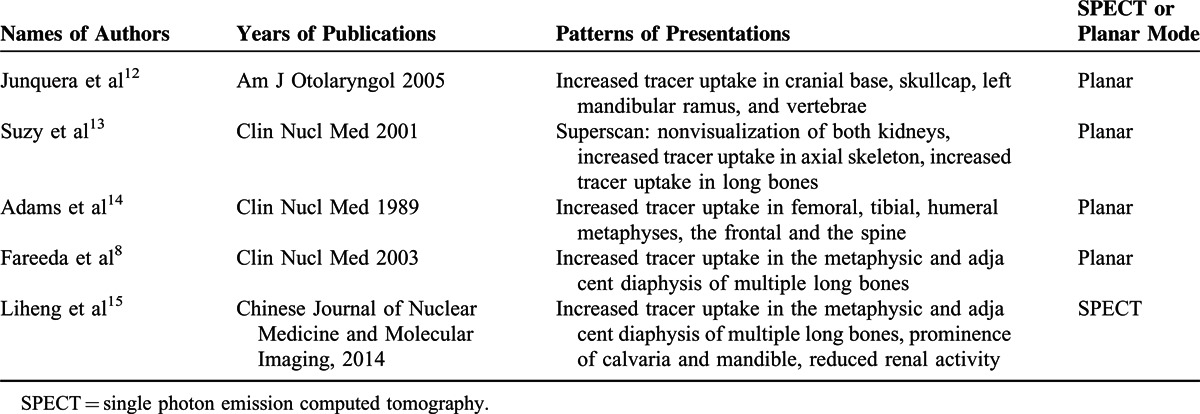
The Literature of “Osteopetrosis and Bone Scan”

## CONCLUSION

Skeletal scintigraphy with ^99^Tc^m^-MDP imaging is helpful to the diagnosis of osteopetrosis. There is characteristic of osteopetrosis different from other bone metabolic diseases, such as intense uptake at the metaphyseal regions of the long bones.
